# Supplementary figures and images for: Degradation graphs reveal hidden proteolytic activity in peptidomes
Source: PLoS Comput Biol. 2026 Feb 20;22(2):e1013972. doi: 10.1371/journal.pcbi.1013972 (PMC12923037; doi:10.1371/journal.pcbi.1013972)

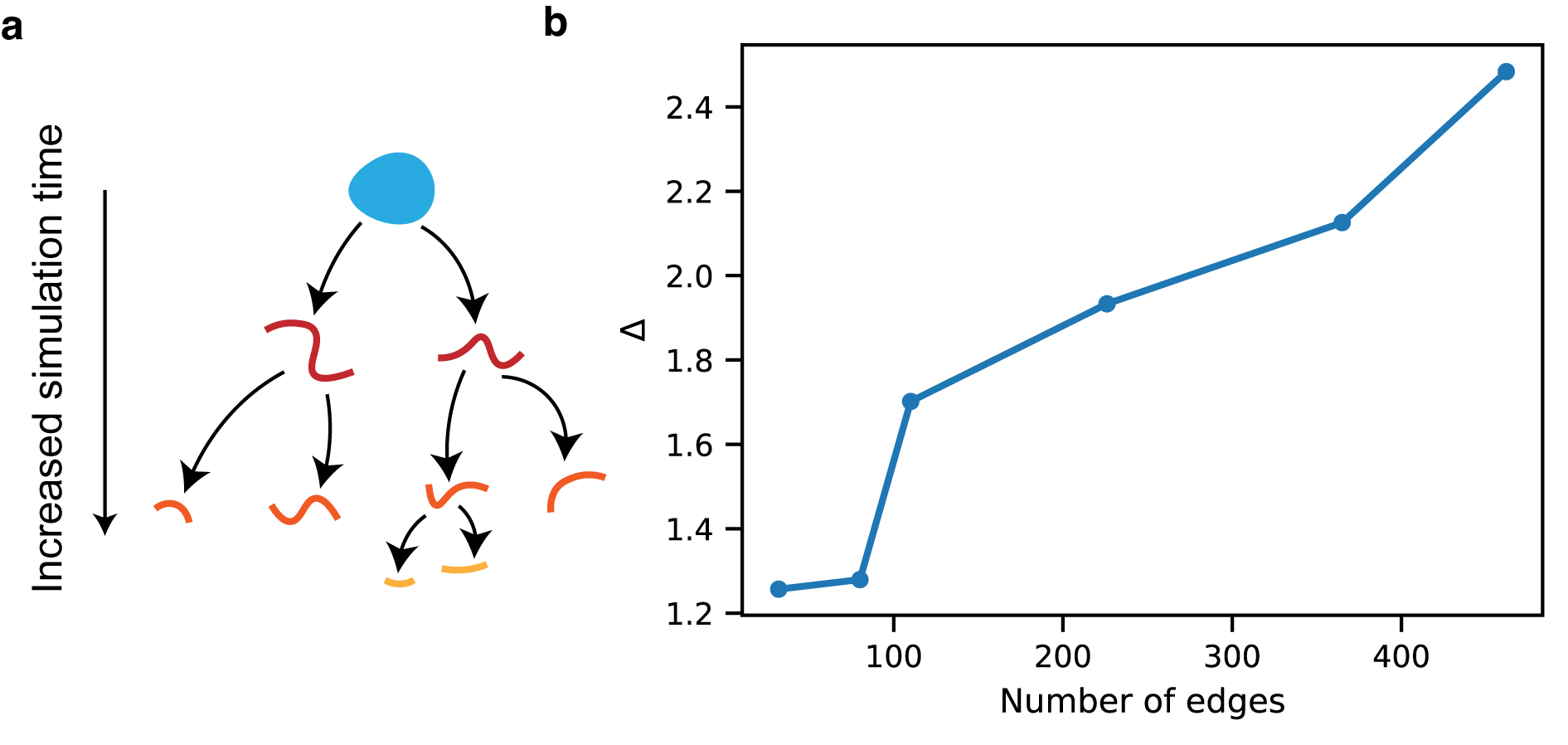

Supplement: S1 Fig — a Schematic illustrating how extended simulation time produces deeper and more branched degradation pathways. b Underestimation ratio (Δ) as a function of graph size, measured by the number of edges generated during simulated proteolysis. As degradation progresses and graphs become larger, Δ increases, reflecting the growing discrepancy between total peptide intensity and the true cumulative degradation flow. (TIFF) [file pcbi.1013972.s001.tif]
