# Supplementary material for: Degradation graphs reveal hidden proteolytic activity in peptidomes
Source: PLoS Comput Biol. 2026 Feb 20;22(2):e1013972. doi: 10.1371/journal.pcbi.1013972 (PMC12923037; doi:10.1371/journal.pcbi.1013972)
Supplement: Attachment — Submitted filename: responses_review1.pdf [file pcbi.1013972.s002.pdf]

We thank the reviewers and the editor for their insightful and constructive comments. We have carefully revised the manuscript to address all concerns and substantially improved both the presentation and scope of the work. The major updates include:

- **Transformation into a full research article.** The manuscript has been largely rewritten to a tighter format and expanded results. The analyses now span both simulated, *in vitro* and *in vivo* datasets, providing a more comprehensive demonstration. As such, figures have been remade and new figures have been added.
- **Clearer articulation of novelty and contribution**. We have restructured the introduction to more explicitly describe how degradation graphs differ from existing degradomic and peptidomic models. We emphasize that this work introduces a new conceptual framework for mechanistic peptidomics, representing one of the first attempts to infer dynamic degradation processes from static peptide data.
- **Expanded analysis**. We now include two new in vivo datasets: urinary peptidomes from diabetic and healthy individuals, and porcine wound fluids infected with *S. aureus* or *P. aeruginosa*. These analyses demonstrate the utility of the degradation graph beyond theory.

We believe that these revisions have strengthened the manuscript and that it now provides a clear and rigorous presentation of degradation graphs as a new tool for peptidomic analysis.

Reviewer #1: I have the following comments.
1. It is recommended that the Introduction section should include an overview of current mainstream protein degradation models, along with an analysis of their differences and improvements compared to the proposed method. Additionally, the core innovations of this study should be clearly articulated in the Introduction.

We have revised the introduction to include an explicit overview of current degradomic and peptidomic approaches, including sequential multi-step models and exoprotease-specific statistical methods. Importantly, we emphasize that this is a new paradigm as few previous studies have attempted to model peptide connectivity or to reconstruct proteolytic networks from static data.

2. The experimental validation is currently limited to simulated data and a single experimental dataset (β-actin), with a relatively small dataset size (only 70 peptides). It is suggested to broaden the validation scope by testing on diverse protein types and larger-scale real datasets.

Two additional experimental datasets have been added: urinary peptidomes from diabetic and healthy individuals and porcine wound fluid peptidomes from bacterial infections. In the diabetes dataset, we study uromodulin which has 86 mapped peptides. In the wound fluid analysis, we study hemoglobin subunit alpha, with 1022 mapped peptides. These analyses showcase that the method can scale and be applied to diverse data. The analyses are now presented in Fig. 5 and 6 in the manuscript.

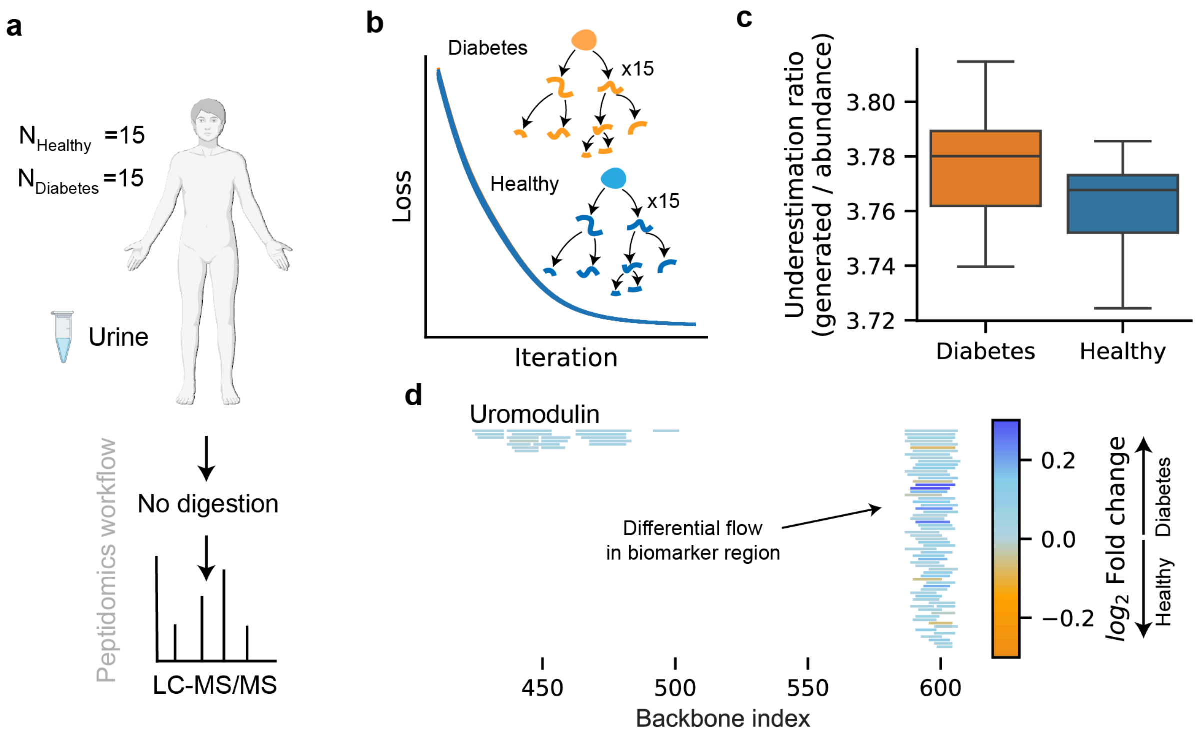

Fig. 5 | Degradation graph analysis of urinary peptidomes from diabetic and healthy individuals. a Peptidomic data from urine samples of individuals with diabetes (n = 15) and healthy controls (n = 15) (54) were analyzed without enzymatic digestion using LC–MS/MS. b For each sample, degradation graphs were reconstructed by gradient descent to fit the modeled peptide distribution to observed abundances. c Comparison of total generated versus observed peptide abundance revealed that conventional quantification underestimated proteolytic activity by approximately 3.7-fold in both groups. d Mapping total inflow along the uromodulin sequence showed a localized increase in degradation flow within the biomarker-associated region, highlighting differential proteolysis between healthy and diabetic samples.

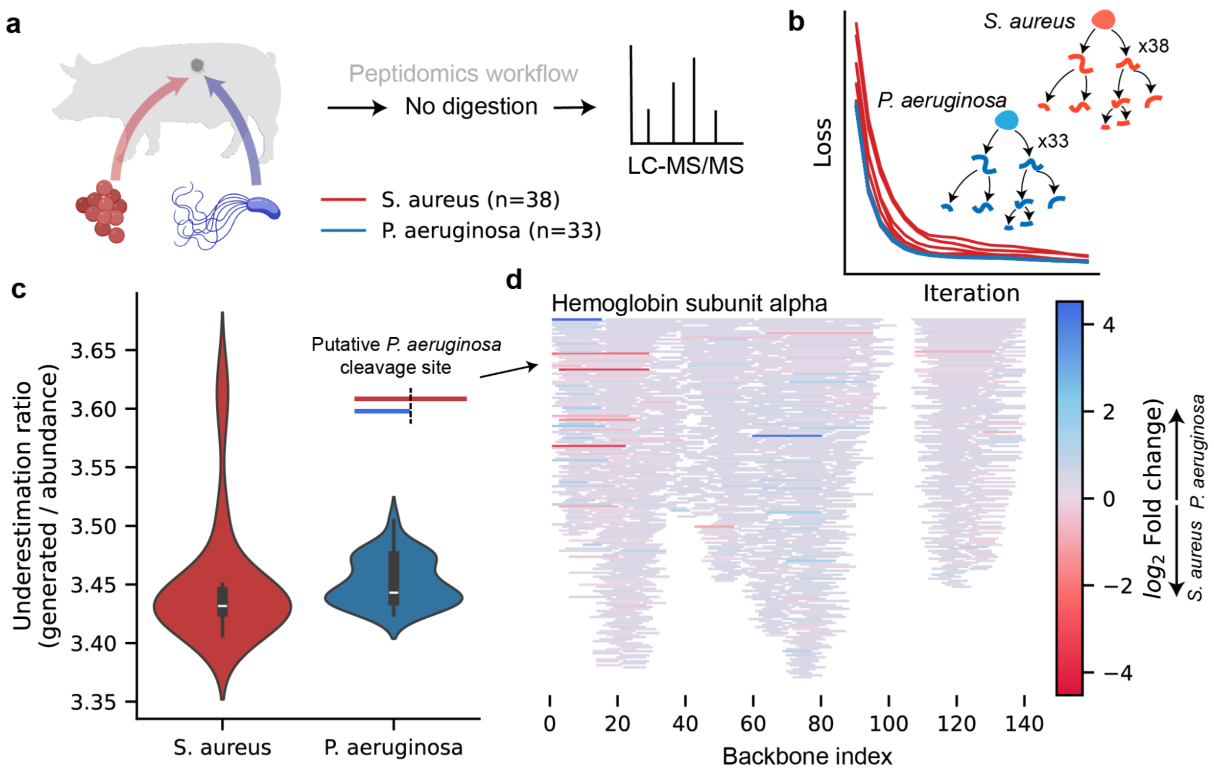

Fig. 6 | Degradation graph analysis of porcine wound fluid peptidomes from bacterial infections. a Peptidomic data from porcine wound fluids infected with Staphylococcus aureus (n=38) or Pseudomonas aeruginosa (n=33) (55) were analyzed without enzymatic digestion using LC–MS/MS. b For each sample, edge transition probabilities were optimized by gradient descent to reproduce measured peptide distributions. c The ratio between generated and observed peptide abundance showed that neglecting sequential degradation led to an average 3.5-fold underestimation of total proteolytic activity. d Visualization of total inflow along the hemoglobin subunit alpha backbone revealed differential degradation flow between infection types, with pronounced variation in the N-terminal biomarker region and an additional differential site around residues 60–80.

3. The paper lacks performance comparisons between the proposed method and existing methods. It is recommended to include relevant benchmark tests to demonstrate its advantages.

At present, no existing computational framework performs equivalent graph-based degradation inference from static peptidomic data. This novelty makes direct benchmarking impossible. However, to highlight the improvement over conventional approaches, we include quantitative comparisons demonstrating that overlooking downstream degradation leads to 3-4x underestimation of proteolytic activity. This discrepancy captures the core advantage of our approach: explicit modeling of degradation connectivity corrects a major bias inherent in current peptidomic quantification and is more true to actual proteolysis.

4. The description of the specific implementation details for optimizing the weights of the degradation graph using gradient descent and linear programming is insufficient. It is suggested to provide algorithm pseudocode or flowcharts to enhance clarity and reproducibility.

We added pseudocode to the main text for both the gradient-based and linear programming algorithms, including input/output definitions and stepwise optimization logic. We have further improved our notation and description of our method in the main text.

Reviewer #2: 1 Incorporate any significant numerical findings within the abstract.

The abstract has been revised to include key numerical findings, notably that overlooking downstream degradation results in an approximately 3-4x underestimation of proteolytic activity across datasets.

2.In the beginning, emphasise the novelties and fresh contributions of your work.
3 Give an overview of earlier research, highlighting what has been accomplished, what is still lacking, and your contributions in relation to those findings.

The introduction now explicitly highlights the conceptual novelty of degradation graphs and situates the method within the broader landscape of degradomics and peptidomics. We discuss prior work on sequential degradation models, their limitations, and how our probabilistic framework generalizes these ideas into a form suitable for modern data-driven analysis.

4 Perform further benchmark analysis to illustrate the benefits or drawbacks of your approach. To assess how much the adoption of your suggested advanced ways improves things, it would be helpful to have more comparisons with some older approaches.

As degradation-graph inference is a new formulation, no comparable algorithm exists for direct benchmarking. To demonstrate the benefits of the approach, we instead evaluate performance on (i) an expanded simulated proteolysis datasets combining multiple proteins. This has been added to Fig. 4. (ii) 2 real peptidomic datasets, one of urinary peptidomes from diabetic and healthy individuals and one of porcine wound fluid peptidomes from bacterial infections. These experiments show that degradation graphs improve interpretability and yield new insights compared to traditional peptide-based analysis. These analyses have been added as Fig. 5 and 6 in the manuscript and are attached below:

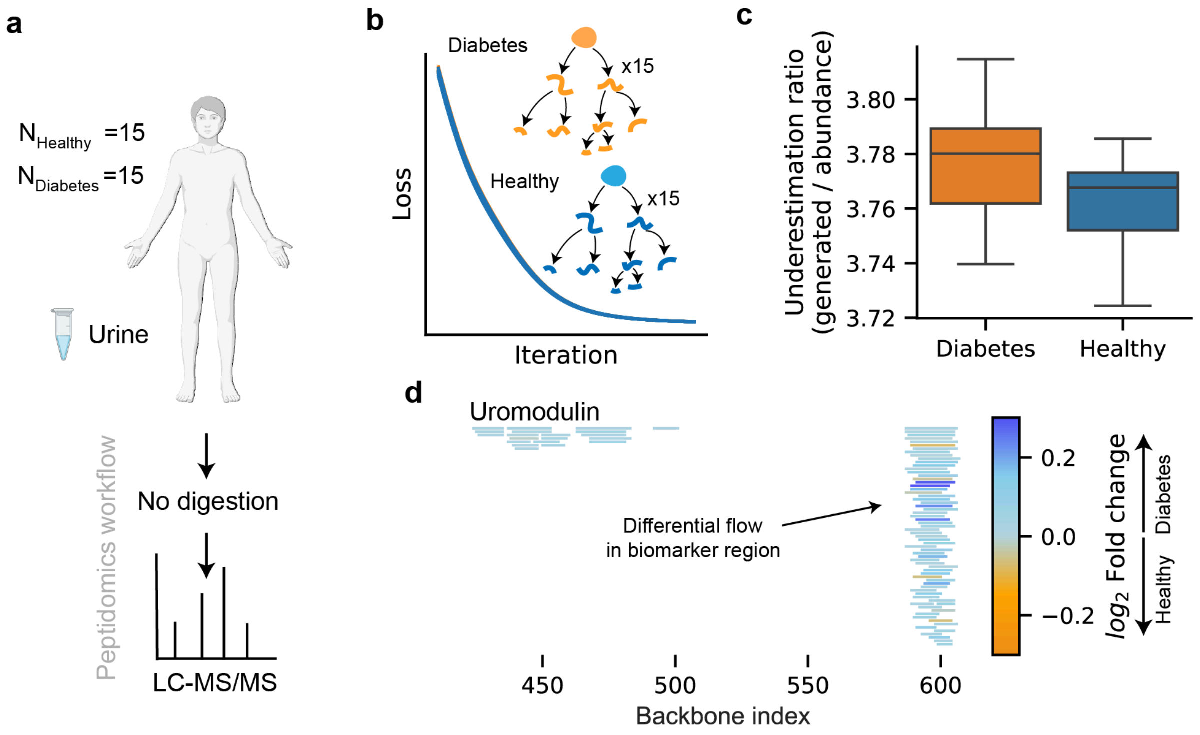

Fig. 5 | Degradation graph analysis of urinary peptidomes from diabetic and healthy individuals. a Peptidomic data from urine samples of individuals with diabetes (n = 15) and healthy controls (n = 15) (54) were analyzed without enzymatic digestion using LC–MS/MS. b For each sample, degradation graphs were reconstructed by gradient descent to fit the modeled peptide

distribution to observed abundances. c Comparison of total generated versus observed peptide abundance revealed that conventional quantification underestimated proteolytic activity by approximately 3.7-fold in both groups. d Mapping total inflow along the uromodulin sequence showed a localized increase in degradation flow within the biomarker-associated region, highlighting differential proteolysis between healthy and diabetic samples.

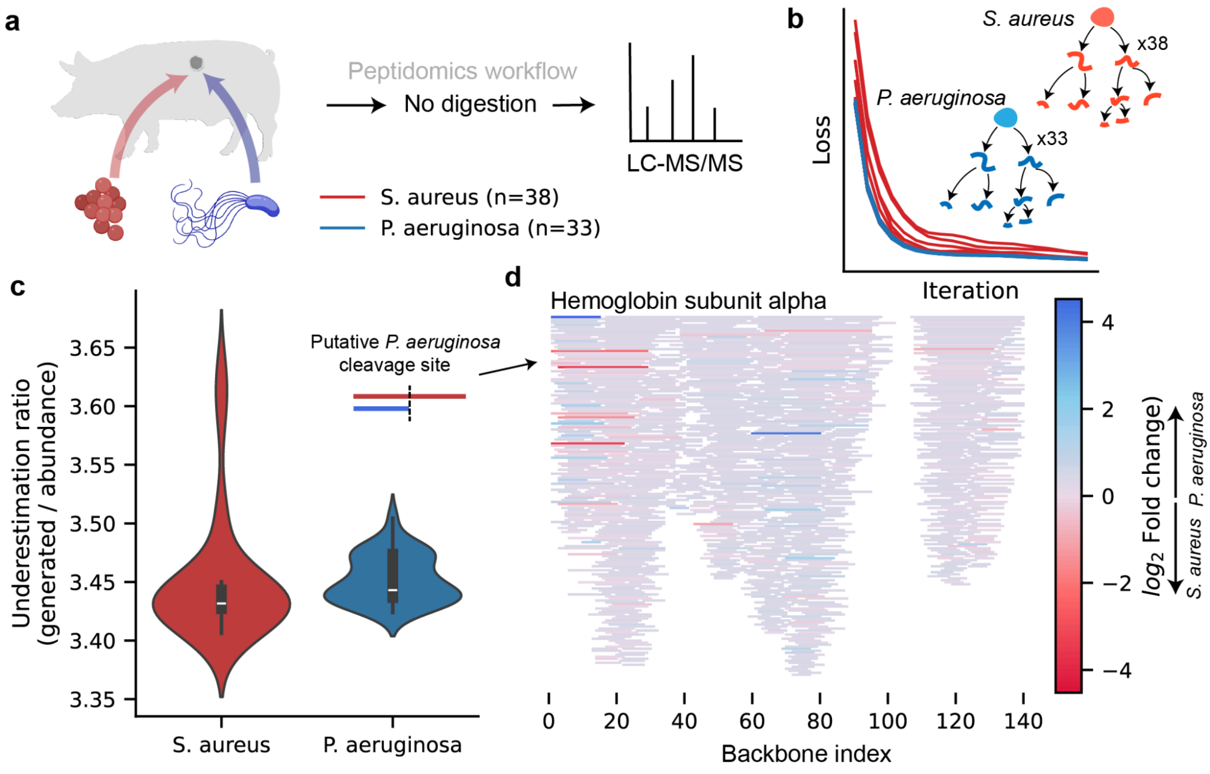

Fig. 6 | Degradation graph analysis of porcine wound fluid peptidomes from bacterial infections. a Peptidomic data from porcine wound fluids infected with Staphylococcus aureus (n=38) or Pseudomonas aeruginosa (n=33) (55) were analyzed without enzymatic digestion using LC–MS/MS. b For each sample, edge transition probabilities were optimized by gradient descent to reproduce measured peptide distributions. c The ratio between generated and observed peptide abundance showed that neglecting sequential degradation led to an average 3.5-fold underestimation of total proteolytic activity. d Visualization of total inflow along the hemoglobin subunit alpha backbone revealed differential degradation flow between infection types, with pronounced variation in the N-terminal biomarker region and an additional differential site around residues 60–80.

5 Please give github website to release the code with datasets.

The implementation is publicly available via GitHub. The repository URL (https://github.com/ErikHartman/degradation-graphs) has been included in the main text.

6 Please perform parameter analysis and abration experiments.

To assess the robustness of degradation graph inference, we systematically varied the learning rate and number of epochs for both uromodulin (~100 peptides) and HBA (~1000 peptides). Across all settings, the underestimation ratio (delta) and final loss (MSE) remained stable, and the top-k graph components showed high reproducibility (Jaccard 0.8–1.0) once the optimization converged. This demonstrates that the method's outputs are robust to parameter choice. While traditional ablation studies are not directly applicable to this modeling framework, our parameter analysis provides strong evidence of stability and convergence reliability.

Uromodulin in diabetes-dataset:

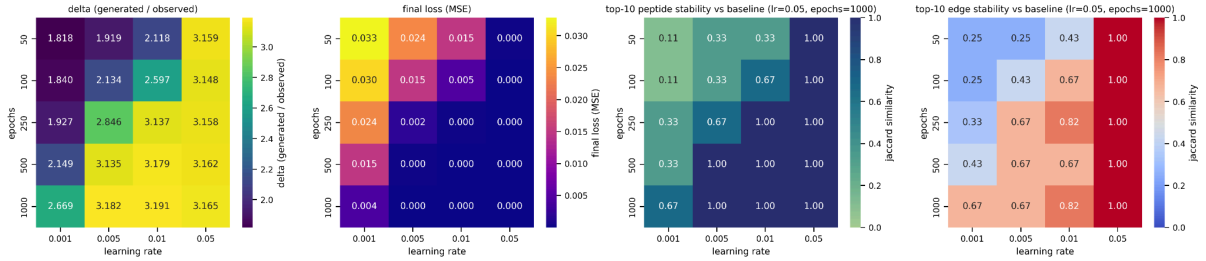

HBA in wound infection-dataset (*P. aeruginosa*):

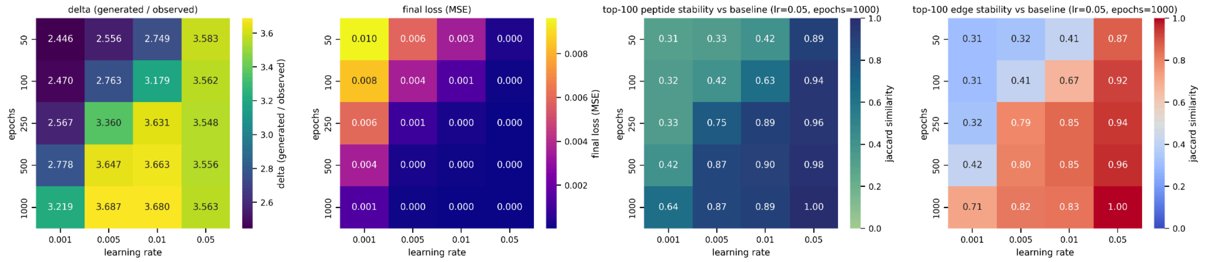

Reviewer #3: In PCOMPBIOL-D-25-01342 entitled "Degradation Graphs for Mechanistic Peptidomic Analysis", authors proposed degradation graphs to representing proteolytic processes from peptidomic data, and show a case about how degradation graphs can be used for prediction of graph types. It should be an interesting work to provide more computational model and biological details of protein and its dynamics in biological system. I have some questions and suggestions:
(1) The main figures should improve and provide more details about the model. For Figure 2a, as the tree structure with root of one protein, all the peptides in the tree should be given variable annotations, which can help understand the relation of variables in the model by Markov chain.

We thank the reviewer for their interest in the method. We have remade many of the main figures to increase clarity. In figure 2, adding variable labels to all nodes cluttered the visualization, however key nodes and edges are now annotated to illustrate how the model variables map to the theoretical definitions.

(2) Similarly, in Figure 3a, it is necessary to illustrate the common and specificity between traditional and graph methods.

We modified figure 3 (now figure 4) to visually contrast conventional peptide-based analysis with graph-based modeling.
(3) It is necessary to provide a pseudo-code for the computational framework in the main text.

Pseudocode for both gradient-based and linear programming methods is now included in the main text.

(4) Authors have provided case application to a small experimental data, which is based on a pool data. It is necessary to provide further deep mechanistic study on larger dataset with control and case samples (e.g. normal and disease samples).

We have expanded validation to include *in vivo* datasets: diabetic vs healthy urine samples and infected wound fluids with *S. aureus* vs *P. aeruginosa*. In both of these cases, we do not pool samples, but instead find degradation graphs of all samples individually. These analyses demonstrate

that degradation graphs recover interpretable differences in degradation flow and accurately identify differential degradation regions in known biomarkers. The results for these analyses are now presented in Fig. 5 and Fig. 6 in the main text, and the figures have also been attached below:

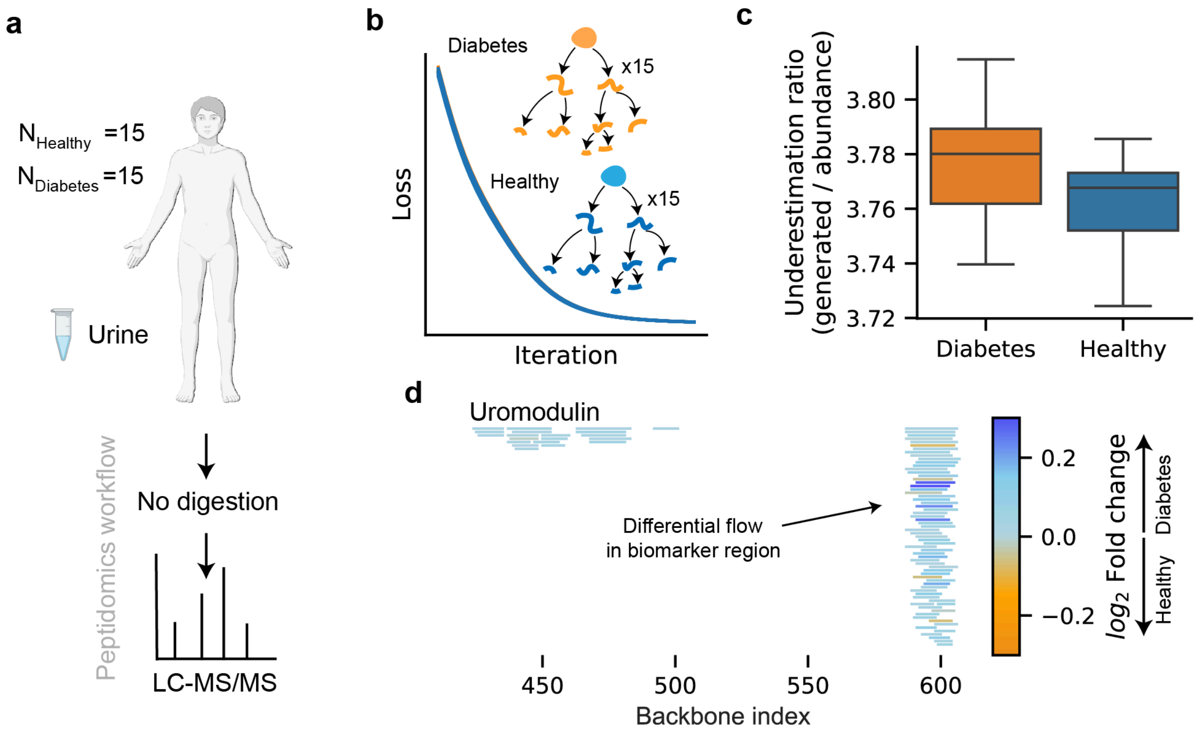

Fig. 5 | Degradation graph analysis of urinary peptidomes from diabetic and healthy individuals. a Peptidomic data from urine samples of individuals with diabetes (n = 15) and healthy controls (n = 15) (54) were analyzed without enzymatic digestion using LC–MS/MS. b For each sample, degradation graphs were reconstructed by gradient descent to fit the modeled peptide distribution to observed abundances. c Comparison of total generated versus observed peptide abundance revealed that conventional quantification underestimated proteolytic activity by approximately 3.7-fold in both groups. d Mapping total inflow along the uromodulin sequence showed a localized increase in degradation flow within the biomarker-associated region, highlighting differential proteolysis between healthy and diabetic samples.

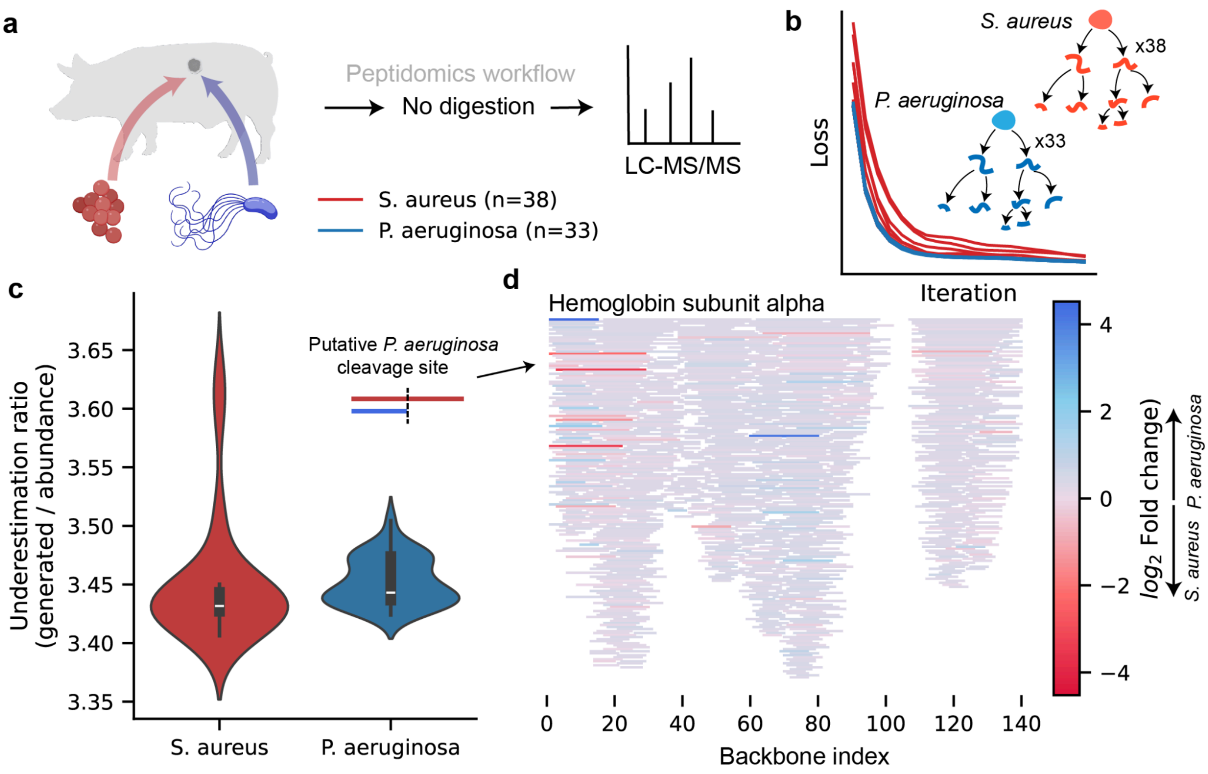

Fig. 6 | Degradation graph analysis of porcine wound fluid peptidomes from bacterial infections. a Peptidomic data from porcine wound fluids infected with Staphylococcus aureus (n=38) or Pseudomonas aeruginosa (n=33) (55) were analyzed without enzymatic digestion using LC–MS/MS. b For each sample, edge transition probabilities were optimized by gradient descent to reproduce measured peptide distributions. c The ratio between generated and observed peptide abundance showed that neglecting sequential degradation led to an average 3.5-fold underestimation of total proteolytic activity. d Visualization of total inflow along the hemoglobin subunit alpha backbone revealed differential degradation flow between infection types, with pronounced variation in the N-terminal biomarker region and an additional differential site around residues 60–80.

(5) It is suggested to provide some tables to summarize the current available data and method for peptidomic study.

We appreciate the suggestion but consider this beyond the scope of the paper, as it has now been re-written as a research article instead of a perspective, which is focused on the introduction and validation of a new modeling framework rather than a comprehensive review.

(6) It is better to provide code and data of this study in public domain.

All code and representative datasets are publicly available on GitHub and referenced in the manuscript.

(7) The manuscript must be proofread to eliminate all spelling errors. Such as in Page 8, “TTo address the challenges …”

The manuscript has been carefully proofread, and all typographical errors (including the one noted on page 8) have been corrected.
