# Supplementary material for: Degradation graphs reveal hidden proteolytic activity in peptidomes
Source: PLoS Comput Biol. 2026 Feb 20;22(2):e1013972. doi: 10.1371/journal.pcbi.1013972 (PMC12923037; doi:10.1371/journal.pcbi.1013972)
Supplement: Attachment — Submitted filename: responses_review_2.pdf [file pcbi.1013972.s003.pdf]

Reviewer #1: I have no other comments.

We thank the reviewer for their constructive comments in prior revisions.

Reviewer #2: The authors addressed most of my prior comments. However I have several comments as follows,
1. Explicitly distinguish degradation graphs from prior sequence-based degradation models (e.g., sequential multi-step reaction models) beyond brief mentions. Highlight unique contributions to graph structure (directed acyclic design, absorption probability) and how they solve unmet needs in dynamic proteolysis inference from static data.

We agree with the reviewer that the novelty of our work compared to prior efforts need to be highlighted further. We have therefore revised the paragraph on prior works in the introduction, to highlight contributions and the added novelty of this work:

"Current data analysis strategies in degradomics and peptidomics treat peptides as static endpoints, although peptides are intermediates that undergo further proteolysis. Only a few studies have attempted to model this process. Yi et al. showed that exoprotease activity can be represented as a sequential multistep reaction using isotopically labelled fibrinogen peptide A and time-resolved quantification of its degradation products (Yi et al., 2008). Kluge et al. introduced the first formal framework for modelling exoprotease activity as a Markov process on a graph in which edges represent cleavage events and parameters correspond to amino acid specific degradation rates (Kluge et al. 2009). Aiche et al. extended this framework to endoproteolytic activity and estimated kinetic parameters from time series data (Aiche et al. 2012). These studies provide a conceptual basis and proof-of-concept for modelling protein degradation but remain restricted to exoproteolysis or depend on temporally resolved measurements that are rarely available. They also precede modern informatics workflows, which limits their applicability to large datasets. As a result, upstream protease activities are systematically underestimated and degradomics and peptidomics remain analytically disconnected."

Further, when deemed appropriate we have contrasted our method to prior works. For example, in the introduction:

"This framework treats peptides as dynamic intermediates and captures the sequential nature of degradation ***without the need for time-series data, making it a natural extension to prior works."***

and in the results:

"In contrast to previous approaches, this method does not require time-resolved measurements and can model protein degradation by applying modern optimization techniques to large peptidomes generated with contemporary mass spectrometry workflows."

2.Strengthen the rationale for choosing both gradient descent and linear programming optimization. Explain trade-offs (e.g., flexibility vs. constraint satisfaction) with empirical comparisons (e.g., convergence speed, stability across datasets) to guide users on method selection.

We agree that this needs to be clarified in the manuscript. While the linear programming approach fell out naturally from the intuitive flow-based interpretation of the degradation graphs, we consider gradient descent to be the most applicable and useful algorithm, since the objective can be modified to fit assumptions about the data and biology by including terms - although we do not delve deeper into additional terms here. We have added the following section on runtime and algorithm motivation:

“Both optimization schemes are implemented in modern frameworks like PyTorch [@pytorch] and PuLP [@pulp] are available in our GitHub repository. The advantage of the LP formulation is fast and guarantees exact satisfaction of the flow constraints. While gradient descent is slower, it is more flexible because the objective is written as a differentiable loss, which allows future extensions such as regularization terms, priors, or custom penalties that guide the solution toward preferred graph structures. This flexibility makes gradient descent well suited for exploring alternative modeling assumptions that cannot be expressed cleanly using linear constraints. In practice, the runtime remains short even for large peptidomes, so the additional compute required for gradient descent is minimal. For this reason, we view gradient descent as the default choice.

Table 1: Runtime of gradient descent and linear programming solvers across increasing graph sizes.

# edges	*gradient descent (s, mean ± std)*	*LP (s, mean ± std)*
24	*1.60 ± 0.26*	*0.031 ± 0.017*
46	*2.59 ± 0.02*	*0.0229 ± 0.0017*
68	*3.79 ± 0.04*	*0.0235 ± 0.0021*
128	*6.22 ± 0.01*	*0.0261 ± 0.0037*
253	*10.97 ± 0.14*	*0.0376 ± 0.0137*
360	*15.95 ± 0.24*	*0.0540 ± 0.0150*

“

3. Address peptide detection bias: Discuss how incomplete peptide coverage (a key limitation) impacts graph inference and propose mitigation strategies (e.g., imputation methods tailored to degradation logic, or sensitivity analyses on missing peptides).

We agree that the peptide detection bias requires careful consideration and warrants more explanation in the article. Beyond our section in the Methods titled *Formalization of key limitations and assumptions,* we have now expanded the discussion to clearly formulate the two main problems that remain to accurately model protein degradation. We have further introduced the idea of imputation tailored to degradation logic in the following section:

“There are two major challenges in building degradation graphs from peptidomic data. [...]

The second challenge is inherited from the field of mass spectrometry peptidomics at large, which is the issue with peptide identification and quantification. Mass spectrometry fails to identify and quantify

peptides for many reasons, including ionization efficiency, length, charge, or low abundance (see Methods: Formalization of key limitations and assumptions). If key intermediates are not detected, we do not have the true peptide distribution, and no method can recover a completely correct graph from incomplete data. Missing peptides can hide real branches in the degradation pathway or create apparent shortcuts that do not occur in biology. However, there are practical developments that could utilize the graph structure to help mitigate this issue. For example, imputation methods designed around degradation logic could infer likely missing intermediates, for example by enforcing that a parent fragment should give rise to two child fragments with compatible abundances. Further, sensitivity analyses where we add plausible missing peptides and examine how the graph changes, could show how strongly the results depend on detection gaps.

The severity of the detection problem will lessen as mass spectrometry instrumentation and workflows continue to improve, expanding the portion of the peptidome that can be reliably measured. The non-uniqueness issue will benefit from growing databases of known protease behavior and cleavage patterns. Together, these developments will make degradation graphs more accurate and more useful for understanding proteolysis in complex samples."

4. Expand the machine learning section: Test the GraphConv model on more proteases (beyond trypsin/elastase) and real-world datasets (not just simulated data) to validate its ability to capture protease-specific signatures in complex samples.

We appreciate the need for further validation of the graph neural networks and their application to degradation graphs. Given the novelty of the field, it is still difficult to find datasets that are large enough to accommodate learning of complex samples, as high complexity requires more data for learning. That is why we resorted to simulated data where we could simulate large sets of samples with high degree of exoprotease activity. However, we have now also added benchmarking on data from lysated cells digested with different enzymes. Although this sample type is simpler than complex clinical samples, it demonstrates the application to real data. We have also added a statement about the proof-of-concept nature of this application in the results. The updated paragraph now reads:

*"Finally, the explicit graph topology enables graph-based inference (Fig. 4d). Node and edge-level features such as position, length, abundance, and transition probability can be integrated into machine learning models that recognize characteristic degradation regimes. As an example, degradation graphs derived from *in silico* proteolysis of several proteins were classified by a GraphConv network, which separated trypsin from elastase-derived graphs with high accuracy (overall ROC–AUC = 0.915, Fig. 4e). We also applied the GraphConv network on cell lysate peptidomes when digested with different enzymes. β-actin was chosen due to its high coverage in the samples, and the graphs were identified through gradient descent (Fig. 4f). On these samples, the model achieves a near perfect ROC-AUC (Fig. 4g). While these demonstrations are limited to simulated data and simple digestions, it demonstrates the concept that degradation graphs not only correct quantification but also provide a foundation for predictive analysis of protease activity."*

and the new Figure 4 is:

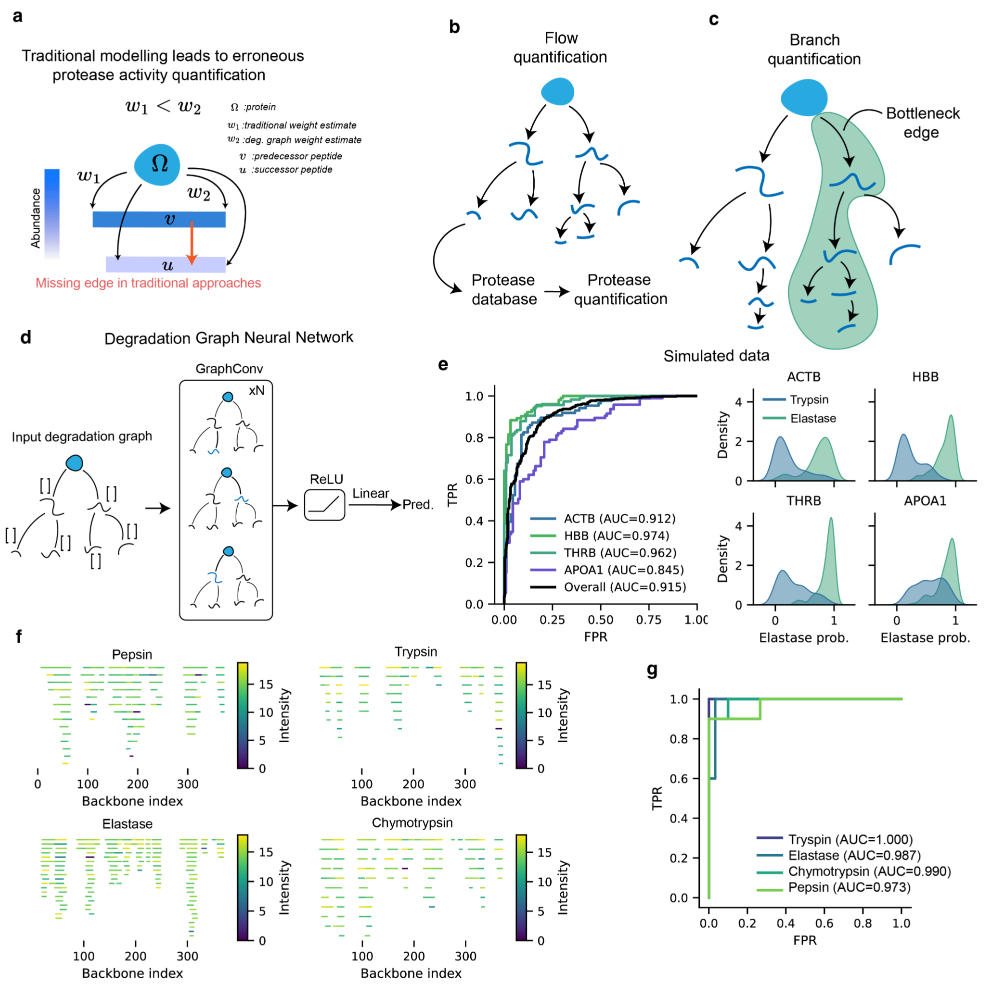

We have also added a statement about the limitations in the discussion:

"Because degradation graphs encode proteolysis as a directed acyclic graph with explicit node and edge features, they can be used directly in graph neural networks and other machine learning models that are designed to learn from graph-structured data, unlike earlier approaches based solely on peptide intensities. In our demonstration, a simple GraphConv architecture could classify degradation graphs with topological and sequence-derived features alone. Although we only present this application as a proof-of-concept, it opens the door to predictive models that learn protease signatures, degradation kinetics, or tissue-specific proteolytic patterns directly from data."

5. Improve figure annotations: For key figures (e.g., Fig. 2, 4), add labels for biological relevance (e.g., "bottleneck peptide linked to inflammation") and clarify how graph features map to experimental observations.

Thank you for this suggestion. We have now added an illustration of the biological origin of the peptide distribution in Fig. 2 and an illustrative example to Fig. 4.

Reviewer #3: Authors have responded to all my concerned questions, and made certain revisions.

There are a few remaining points:
(1) Fig3f, the numbers in x-axis should be labeled clearly.

The x-axis has now been labeled clearly.

(2) Fig5c, there should be P value of significance test of group differences between Diabetes and Healthy. (3) Fig6c, there should be P value of significance test of group differences between S.aureus and P.aeruginosa.

We do not believe formal significance testing is appropriate in this context because the absolute differences between groups are small, and the sample sizes are not large enough to support reliable statistical inference. Although a nominally significant P value can be obtained for the *S. aureus* vs *P. aeruginosa* comparison, this result is likely driven by sampling noise rather than a meaningful biological effect. We have therefore added clarifying statements in the manuscript noting that no statistically robust differences were observed between the groups in Fig. 5c or Fig. 6c.

Reviewer #4: This study achieves significant breakthroughs in the analysis of protein dynamic degradation by constructing an innovative computational framework called the 'degradation graph.' For the first time, this method models protein degradation as a directed acyclic graph (DAG) and introduces the probabilistic flow analysis paradigm (Figures 2a–c), successfully addressing the systematic underestimation problem in traditional proteomics analyses caused by neglecting the order of degradation. By proposing the dual concepts of 'absorption probability' and 'flow quantification,' combined with parameter sensitivity analysis and a multi-dimensional validation system (simulated data in Figure 4, in vitro β-actin data in Figure 3, and diabetic urine and infected wound fluid data in Figures 5–6), the method significantly improves the quantification accuracy of protease activity. Notably, during the revision process, the authors added two critical clinical datasets on diabetes and wound infection and supplemented technical details such as algorithm pseudocode, effectively addressing the initial review comments.

We thank the reviewer for the positive and thoughtful evaluation, and appreciate the recognition of the conceptual contributions and the improvements made during revision.

Based on the current quality of the manuscript, it is recommended to address the following issues before acceptance:

1. Although the authors state on page 8 that “there is currently no directly comparable computational framework,” the manuscript lacks a systematic comparison with any simplified baseline methods, making it difficult to intuitively demonstrate the quantitative advantage of the degradation graph. Please include in the supplementary materials a comparison experiment with the conventional "peptide intensity summation method," i.e., directly using observed peptide abundances as protease activity estimates and comparing with the total flow estimates from the degradation graph, further quantifying the "3–4 fold underestimation" mentioned in Figures 3f, 5c, and 6c.

We have now clarified the quantitative advantage of using degradation graphs when it comes to estimation of proteolytic activity in the results-section titled “Why degradation graphs matter”, further clarifying the underestimation ratio. We have further performed an analysis to see how the underestimation ratio (delta) varies with peptidome size, by simulating degradation for different amounts of time. This revealed that the underestimation ratio increases with the graph size, which is expected because larger graphs contain longer degradation pathways, and summing observed peptide intensities ignores the cumulative loss of abundance along these paths. This demonstrates that conventional peptide-summation approaches increasingly underestimate proteolytic activity as the complexity of the peptidome grows. The following section has now been added to the results alongside a supplementary figure:

“We examined how the underestimation ratio changes as a function of degradation extent in a proteolysis simulation. As simulation time increased and more cleavages accumulated, the ratio rose (Supplementary Fig. 1). This behavior is expected, as larger degradation graphs contain longer and more branched pathways, and simply summing observed peptide intensities fails to capture the cumulative loss of abundance along these routes. These results show quantitatively that conventional peptide-summation methods increasingly underestimate proteolytic activity as the peptidome becomes more complex.”

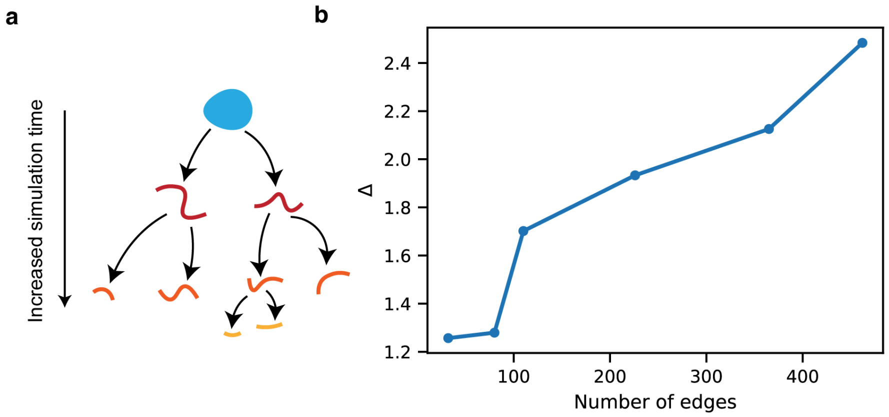

2. Page 18 mentions the problem of "Unidentifiability," but the stability of the model under different initializations or noisy conditions is not systematically evaluated. Please show this as much as possible in the methods or figures, such as the coefficient of variation of edge weights from multiple runs of gradient descent on the same dataset.

We agree with the reviewer that the problem of unidentifiability needs to be evaluated. We therefore ran gradient descent from different random initializations (five replicates) across graphs of increasing size and measured variation in edge weights using their coefficient of variation, as suggested.

We found that the variation increased with peptidome size, but remained low, as the mean coefficient of variation remained below 10% in the tested interval. The following figure is now included as Fig. 3d, alongside the following section in the results:

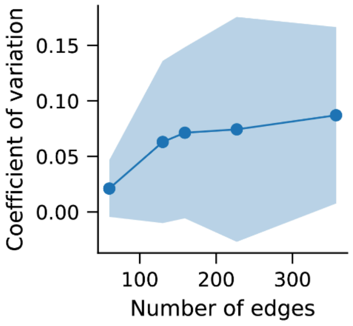

*“The identification of a degradation graph is an underdetermined problem because different graph structures can produce the same observed peptide distribution (see **Discussion** and **Methods: Formalization of key limitations and assumptions**). To assess how much the inferred graphs vary, we ran gradient descent from different random initializations (five replicates) across graphs of increasing size. We measured variation in edge weights using their coefficient of variation. Although the mean variation increased with graph size, it remained below 10 percent even for the largest graphs (Fig. 3d).”*

We further added an analysis of the biological interpretation of the outcomes for the analyzed peptidomes (UMOD and HBA) as a new figure. In this analysis, we varied the learning rate and epochs to generate different degradation graphs of these peptidomes, and investigated the underestimation ratio (delta), as well as the stability of the top peptides and top edges, as measured by total inflow and edge weight respectively. These results show that although we have variation in the resulting graphs, the general outcomes are consistent. The following figure and paragraph were added to the results section:

"We tested how sensitive the method is to training settings by sweeping over learning rates and numbers of epochs when applying gradient descent on a random sample from the clinical datasets. For both datasets and peptidomes, when the loss converged, the underestimation ratio (Δ) increased and stabilized. The peptides with the highest inflow and the edges with the strongest weights were recovered consistently across runs, especially in the smaller UMOD graph. The larger HBA graph showed more variation but still produced stable top features once training had converged (Fig. 7a,b)."

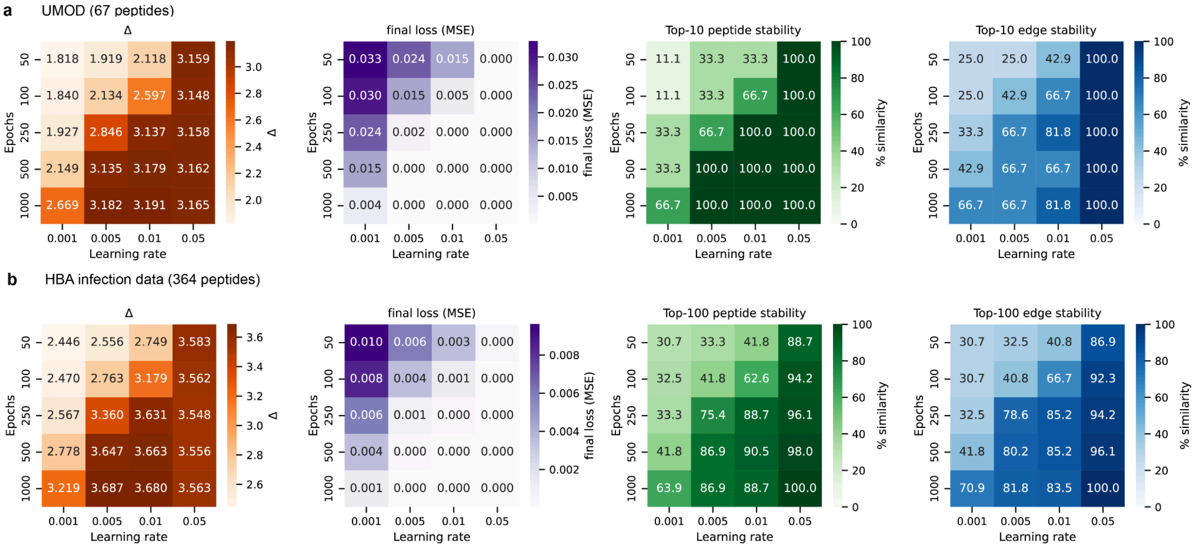

3. Please adjust the clarity of textual content in the figures and use vector graphics wherever possible.

All figures were prepared and submitted in vector format. Any apparent reduction in quality is likely due to conversion by the online submission system rather than limitations in the source files. We will ensure that the final production files maintain full vector resolution.

This study is highly innovative and practical in terms of conceptual, methodological, and application aspects, has adequately addressed the reviewer comments from the initial draft, and has made significant progress in both theoretical and experimental validation.

I recommend revisions before acceptance, mainly addressing the three points mentioned above to provide further clarification or analysis, thereby enhancing the rigor and persuasiveness of the manuscript.

We thank the reviewer for their constructive comments!
